# Palliative care education in the undergraduate medical curricula: students’ views on the importance of, their confidence in, and knowledge of palliative care

**DOI:** 10.1186/s12904-019-0458-x

**Published:** 2019-08-28

**Authors:** Jolien Pieters, Diana H. J. M. Dolmans, Daniëlle M. L. Verstegen, Franca C. Warmenhoven, Annemie M. Courtens, Marieke H. J. van den Beuken-van Everdingen

**Affiliations:** 1 Department of Educational Development and Research Faculty of Health, Medicine and Life Sciences, Maastricht University, Universiteitssingel 60, 6229 ER Maastricht, The Netherlands; 2 Centre of Expertise for Palliative Care, Maastricht UMC+, Maastricht, The Netherlands

**Keywords:** Undergraduate medical education, Students, Palliative care

## Abstract

**Background:**

The need for palliative care is increasing. Since almost every junior doctor will come across palliative care patients, it is important to include palliative care in the undergraduate curriculum. The objective of this research is to gather undergraduate students’ views on palliative care in terms of its importance, their confidence in and knowledge of the domain.

**Methods:**

Final-year medical students at four Dutch medical faculties were surveyed. The questionnaire measured their views on the education they had received, their self-reported confidence in dealing with palliative care patients and their knowledge of palliative care.

**Results:**

Two hundred twenty-two medical students participated in this study. Students considered palliative care education relevant, especially training in patient-oriented care and communication with the patient. Students felt that several topics were inadequately covered in the curriculum. Overall, the students did not feel confident in providing palliative care (59.6%), especially in dealing with the spiritual aspect of palliative care (77%). The knowledge test shows that only 48% of the students answered more than half of the questions correctly.

**Conclusion:**

The students in this study are nearly junior doctors who will soon have to care for palliative patients. Although they think that palliative care is important, in their opinion the curriculum did not cover many important aspects, a perception that is also in line with their lack of confidence and knowledge in this domain. Therefore, it is important to improve palliative care education in the medical curriculum.

## Background

Due to the aging population and growing number of people with chronic diseases, such as heart failure, Chronic Obstructive Pulmonary Disease (COPD), and dementia [1, 2] and the higher survival rate for diseases like cancer, the number of patients needing palliative care due to life-threatening illness is increasing. The World Health Organization (WHO) defines palliative care as ‘an approach that improves the quality of life of patients and their families who are facing problems associated with life-threatening illness through the prevention and relief of suffering by means of early identification and impeccable assessment and treatment of pain and other problems, physical, psychosocial and spiritual [3]‘. Providing palliative care is challenging because of the multidimensional (physical, psychological, social and spiritual) and multidisciplinary aspects involved. Physicians working in nearly all specialties and many care settings regularly have to provide palliative care to the chronically and terminally ill. They should, therefore, acquire in their training the necessary attitudes, knowledge, and skills to do so [4].

The World Health Organization [5] and the European Association for Palliative Care [6] (EAPC) underline the importance of palliative care education. Lack of palliative care education in the undergraduate medical curricula throughout Europe is considered as one of the most important barriers in the integration of palliative care in health care systems [7]. Several studies have reported that medical students are given inadequate education and training in palliative care [8–10] and many junior doctors across different countries (for example: The United States [11]; Brazil [12]; Germany [13] and Turkey [14]) do not feel well prepared. The majority of medical graduates reported that they felt uncertain about providing palliative care [13]. They lack knowledge of palliative care [13], especially on the pain and symptom control aspects [13]. However, research shows that an integrated palliative care curriculum leads to increasing knowledge [15], and physicians with special education in palliative medicine make less aggressive decisions in end-of-life care, as to withdraw life-prolonging therapies, indicating the importance of palliative care education [16].

Currently, in the Netherlands, undergraduate medical curricula spend little time on palliative care. An EAPC expert committee evaluated the degree to which palliative care education was covered in undergraduate medical curricula in Europe and demonstrated that the Netherlands belongs to the five European countries that scored the lowest [17]. Dutch medical education tends to disregard the competencies required to decide whether or not to treat [18]. A recent study showed that End-of-Life Care education is insufficiently mentioned in the Dutch national blueprint for undergraduate medical curricula and is not fully integrated in compulsory medical curricula at the universities [19]. Another study [20] also demonstrated that, from the perception of undergraduate medical students, more attention should be paid to end-of-life care. Nevertheless, there is an increased interest in palliative care teaching [4, 21]. In order to develop palliative care training that is aligned with the perspectives and needs of undergraduate medical students, it is important to understand how medical students relate to palliative care and what their learning needs are. The perspectives of students on palliative care, as defined by the WHO, are currently unknown and need further research. The objective of this study is to investigate undergraduate students’ views on the importance of education in palliative care, their opinion of the education they have received, and their (self-reported) confidence in and knowledge of palliative care.

## Methods

### Setting and participants

Dutch medical education lasts six years and consists of a three-year bachelor’s program and a three-year master’s program. In the bachelor’s program, students learn the theoretical basics. The master’s program focuses on the application of knowledge in practice. Final-year students were recruited from four of the eight medical universities in the Netherlands to answer the online questionnaire administered for this study.

### Material

The questionnaire used in this research is based on the work of Weber et al. [13] (permission granted). The questionnaire contained three components. The knowledge part is based on the earlier validated Palliative Care Examination by Weissman et al. [22] Cronbach’s alpha coefficient was used to assess the internal consistency within the other components [23]. The other components, the students’ perception of the importance of palliative care education(α = 0.822), their opinion of the education they had received (α = 0.845), and confidence felt in dealing with the various aspects of palliative care (α =0.844), showed that the internal consistency between the items within the different subparts is very good [24]. No items were deleted.

#### Perceived importance and education received

The first part of the questionnaire was based on a translation of the questionnaire by Weber et al. [13] and was elaborated with 11 statements concerning differed aspects of palliative care added by the authors. Participants were asked to evaluate these 11 aspects in two ways (see Table 1). First, they were asked to indicate how important these aspects are and second to what extent had these aspects been covered in their curriculum. They survey employed a five-point Likert scale (1 = not/ barely important or not/ barely covered in the curricula; 3 = neutral; 5 = very important or extensively covered in the curriculum). The section ended with one open question: “Did your education in palliative care miss out on anything?”

**Table 1:** Students’ views on palliative care education and perception of its importance (*N* students = 222) (Scale 1–5; 1 = Lowest Score; 5 = Highest Score)

Question	Perceived importance: mean (SD)	Education received: mean (SD)
Definition of palliative care	4.33 (0.64)	3.90 (0.72)
Patient-focused work with palliative care patients	4.36 (0.68)	3.35 (0.91)
Knowledge of symptom control in palliative patients	4.31 (0.62)	3.10 (0.89)
Communication with palliative care patient and their care system	4.34 (0.73)	2.77 (0.99)
Psychosocial and spiritual needs of the patient	3.55 (0.98)	2.57 (0.10)
Knowing what kind of care is available for palliative patients and who plays a role in it	4.15 (0.69)	3.10 (0.48)
Can work with various healthcare providers in the care of palliative patients	3.82 (0.82)	2.85 (0.93)
Ethical issues concerned with the end of life	3.89 (0.77)	3.95 (0.90)
Grief and loss	3.75 (0.77)	2.84 (0.94)
Reflection on own ideas about death and dying	3.40 (0.97)	2.79 (1.09)
Self-care for physicians in providing palliative care	3.79 (0.83)	2.31 (0.86)

#### Self- reported confidence

This part was also based on a translation of the questionnaire by Weber et al. [13] Students were asked to report their confidence level with regard to ten situations in which palliative care was required (see Table 2). The situations covered the four dimensions (psychosocial, somatic, and spiritual) of palliative care. The degree of confidence was differentiated on a four-point Likert scale (1 = very confident; 2 = confident; 3 = unconfident; 4 = very unconfident).

**Table 2:** Self-reported confidence in the domains of palliative care (*N* students = 213)

Domains (*N* = number of items)	Non-confident % (score 1–2)	Confident %
Integrating the psychological aspects of treating and supervising severely ill and dying patients, I feel …	57.3	42.7
Communicating with severely ill and dying patients, I feel …	38.5	61.5
When explaining to a patient that their disease is incurable, I feel …	56.8	43.2
When explaining to a patient that their tumor-specific treatment (e.g. chemotherapy) will be changed to palliative care, I feel …	50.7	49.3
Treating and guiding terminally ill and dying patients, I feel …	38.5	60.5
Assessing and examining patients with cancer pain, I feel …	27.2	72.8
The basic principles and contents of palliative care, I feel...	43.7	56.3
Treating symptoms that might occur in advanced cancer, I feel...	54.9	45.1
Treating cancer pain, I feel...	46.5	53.5
Integrating the spiritual aspects of treating and guiding severely ill and dying patients, I feel...	77.0	23.0
Overall	59.6	40.4

#### Knowledge

Weber et al [13] based this part of their questionnaire on the Palliative Care Examination by Weissman et al. [22] Their shortened questionnaire comprised eight brief case studies with 21 multiple-choice questions (MCQs). The questions related to pain management (57%), symptoms other than pain (19%), psychosocial issues (19%) and ethics (5%). Each MCQ had five answer options, of which one was ‘I don’t know’. For this study, a palliative care expert (*MvdB*) translated and adapted the Weber et al [13] version to the Dutch context.

### Procedure

A pilot study with five sixth-year medical students was conducted. The students filled out the questionnaire to establish the comprehensibility, clarity of the questions, handling and, duration. No further changes were needed. Then the link to the online questionnaire was sent to the participants. The recruitment of students for this study differed per university. At two universities, a staff member sent the invitation email to last-year medical students. At the two other universities, the students were invited in person by the first researcher, during one of their classes. If they agreed to participate, they received the same email as the students of the first two faculties. The difference in approach is due to faculty-specific rules about conducting research.

The first page of the online questionnaire asked the students for informed consent. The questionnaire was completed anonymously and on a voluntary basis. At the end, students could voluntarily fill in their email address to participate in a lottery for five book vouchers worth €15 each. Their email addresses were not linked to the questionnaire. Non-responders received one reminder.

### Statistical data processing

SPSS 21.0 was used for the statistical analysis. To obtain descriptive results, the data were analyzed in line with Weber et al. [13] Students indicated their perceived importance and education received on a five-point scale. Mean scores per item (*N* = 11) across all students were computed. A score below 3.0 was considered unimportant or insufficiently covered, a score of 4.0 or higher as important or sufficiently covered, and all scores in between were considered to be neutral.

For the self- reported confidence levels and the knowledge test, scores were calculated within answer categories. The scores for the self-reported confidence were calculated in percentages and coded in two categories: ‘(rather) confident’ (score 1–2) and ‘(rather) non-confident (score 3–4)’.

For the knowledge test, the scores were calculated in percentages and coded in three categories: ‘correct answers’, ‘incorrect answers’ and the ‘I don’t know answers’. The categories for the knowledge test were: total score, pain knowledge (questions 1–12), psychosocial knowledge (questions 13, 14, 20, and 21), knowledge about ethics (question 15), and non-pain symptom control knowledge (questions 16–19).

## Results

In total 222 (response rate 38%) final-year students from four Dutch medical universities participated. The mean age was 24.9 years (SD = 1.79) and 160 students (72.1%) were female.

### Perceived importance and education received

The participants scored the importance of education on 11 items describing different aspects of palliative care (Table 1). Overall, students indicated that it is important to incorporate palliative care in their education (57.7%). The scores varied between 3.40 and 4.36 (scale: 1–5). Of the 11 items, five items scored higher than 4.0 (important). The other items scored between 3.40 and 3.90 (neutral). No item scored lower than 3.0 (unimportant). Students indicated that especially ‘patient-focused work with palliative care patients’ (Mean = 4.36, SD = 0.68) and ‘communication with palliative care patients and their care system’ (Mean = 4.34, SD = 0.73) were the most important aspects to learn about. The aspects that scored lowest were ‘reflecting on their own ideas about death and dying’ (Mean = 3.40, SD = 0.97) and the ‘psychosocial and spiritual needs of the patients’ (Mean = 3.55, SD = 0.98).

One hundred students answered the open-ended question “Did your education in palliative care miss out on anything?” Overall, the respondents said that palliative care education did not receive enough attention. Furthermore, they said that the curriculum missed out on treatment options and aspects of self-care for physicians.

The 11 statements were also scored on education received (Table 1). Overall, the students judged their palliative care education as inadequate (45.5%) or were neutral about it (48.9%). The scores on the palliative care education received varied between 2.31 and 3.95 (scale 1–5). Of the 11 items, six scored less than 3.0 (inadequately covered). The other items scored between 3.0 and 3.9 (Neutral). No item scored higher than 4.0 (adequately covered). The students indicated that the most of the education they received concerned ethical issues around end of life (Mean = 3.95, SD = 0.90) and the definition of palliative care (Mean = 3.90, SD = 0.72). Self-care for physicians in providing palliative care (Mean = 2.31, SD = 0.86), the psychosocial and spiritual needs of patients (Mean = 2.57, SD = 0.10) and communication with palliative care patients and their care system (Mean = 2.77, SD = 0.99) scored lowest (Table 1).

### Self-reported confidence

Of the 222 respondents, nine did not complete this self-assessment part of the questionnaire. Table 2 lists their answers. Overall, the students do not feel confident in providing palliative care (59.6%). They stated that they feel most confident in assessing and examining patients with cancer pain (72.8%) and in communicating with severely ill and dying patients (61.5%). They reported feeling least confident about integrating the spiritual aspects (23.0%) and psychological aspects (42.7%) of the treatment and supervision of severely ill and dying patients. Also, they do not feel confident about explaining to the patient that their disease is incurable (43.2%).

### Knowledge

The last part of the questionnaire comprised 21 questions exploring the students’ knowledge of palliative care. Of the 222 respondents, 154 (69.4%) filled in this part of the questionnaire. Approximately half (47.8%) answered more than half of the questions (11 or more) correctly. Between 45 and 55% of the students gave correct answers in the domains of pain knowledge, psychosocial knowledge, and non-pain symptom control knowledge, while 84% of the students gave the correct answer to the question on knowledge about ethics (Table 3). The question answered correctly most often was on communicating the prognosis and the question answered correctly least often was on the side effects of opioids (Appendix). Only 7% of the students gave the right answer to this question.

**Table 3:** Knowledge extrapolated in relation to four topic scores (%) (*N* students = 154)

	Correct answer	Wrong answer	Unsure	N items
Pain management	44.8	33.9	21.3	12
Symptom control	45.6	34.4	20.0	4
Psychosocial	55.5	39.8	4.7	4
Ethics	83.8	16.2	0.0	1

## Discussion

This study demonstrates that medical students view palliative care as an important topic to be covered in their undergraduate medical education. However, according to the surveyed students, most aspects of palliative care are not covered properly. They do not feel confident in providing palliative care and lack the necessary knowledge. These results are in line with to the growing body of research showing that medical students are not adequately prepared to provide palliative care [8, 11–13]. The findings of our study are in line with results reported in various countries like the United States [11] and Germany [13] .

Looking at the various components of the questionnaire, some results are noteworthy. First of all, the respondents indicated that communication in palliative care is one of the most important aspects to learn about, but not much attention is paid to these specific communication skills in their training program. This may explain their rather low level of confidence on communication items*.*

Second, the students indicate that they lack education on the psychosocial and spiritual needs of palliative care patients, which again may explain why they lack confidence in this. This is in line with the research of Best et al. [25] and Ellis et al [26] who found that doctors only rarely raise spiritual aspects of care, although many patients would be interested in discussing issues around spirituality in the medical consultation. Best et al [27] found that the underlying confusion regarding the differences between religion and spirituality is one of the important contributors to the reluctance of doctors to discuss spirituality with patients. Medical curricula are mainly based on the bio-psycho-social model [28], and shifting towards a biopsychosocial-spiritual model could lead to more holistic and better patient care.

Third, students responded positively on the topic of ethics concerned with the end of life care and they indicated that ethical aspects were properly covered. This finding is further supported by the results of the knowledge test, where students scored remarkably high on the ethics question. This result is line with research of Hesselink et al [20] who found that medical students perceived the content and quality of end-of-life- care moderate-to-good. However, Thurn and Anneser [29] reported that German medical students experience moral distress frequently in end-of-life care and emphasize the importance of empowering students by means of training to address their moral concerns. Differences between our findings in which ethics were more positively rated, could be explained by cultural differences or by differences in training.

Although we did not formulate a research question about the relation between the different variables, we correlated the measures to check how they relate to each other. This resulted in a positive correlation between self-confidence and knowledge (R = 0.22; *P* = 0.000) and between self-confidence and the degree to which relevant topics were covered in the curriculum (R = 0.40, *P* = 0.006); which is in line with our expectations; i.e. a better score on knowledge is positively related to self-confidence and a higher score on topics covered during training is also positively related to self-confidence.

Several issues should be taken into account when interpreting the results of this study. This is a multi-center study and the four participating universities are spread geographically throughout the Netherlands. The demographic characteristics of the participating students are comparable to those of the population of Dutch undergraduate medical students. The questionnaire consists of three different components: [1] perceived importance, [2] degree to which a given topic is covered in the curriculum and [3] the degree to which the student feels competent/confident. Put together, the results offer a broad perspective on students’ views and current learning needs.

The study also has some limitations. Just like in the study by Weber et al [13], the response rate was relatively low, which could imply that only motivated students responded [30]. As a consequence, the importance of palliative care education could be overrated. The questionnaire was based on an existing questionnaire in order to connect with the literature. The disadvantage was that this questionnaire pays unequal attention to the different dimensions of palliative care and focuses more on cancer than on other palliative conditions. Another limitation of the study is that only the knowledge section of the used instrument makes use of a formerly validated instrument. The other two sections and the translation of the knowledge section were not revalidated.

This study determined which aspects of palliative care are important to medical students. The results have important implications for practice and research. Universities can use these results to anticipate the learning needs of the students and improve their education programs to better prepare students for a care context in which the need for palliative care will increase due to the expanding aging population and the growing number of people with chronic diseases. Thereby, this study offers an important first step in the development of palliative care education in undergraduate medical curricula. To develop a full picture of what should be covered in the medical curricula will require additional studies with stakeholders other than students involved.

## Conclusions

This study reveals that medical students view palliative care as an important subject that should be addressed properly in their medical education, especially the aspects of patient-focused work and communication. The respondents reported limited confidence in providing palliative care. The four medical schools involved in this study address many palliative care topics to a limited extent in their curricula. The findings will be of interest to all those concerned with the development of palliative care education.

## Data Availability

The datasets used and/or analyzed during the current study are available from the corresponding author on reasonable request.

## Appendix

**Table 4:** Answers to 21 questions exploring knowledge (%) in responding students (*N* students = 154)

	Correct	Wrong	Don’t know
Identify somatic pain	70.8	23.4	5.8
Move to strong opioid	64.3	30.5	5.2
NSAID for bone pain	67.5	11.7	20.8
Treatment of breakthrough pain	37.0	32.5	30.5
Conversion oral to IV morphine	22.7	9.8	67.5
Time to therapeutic for fentanyl patch	27.3	38.9	33.8
Equianalgesic dose of fentanyl	19.5	13.6	66.9
Opioid nausea resolves in < 7 days	35.1	58.4	6.5
Side effects opioids	6.5	89.0	4.5
Worsened pain is worsening cancer	38.3	59.8	1.9
Identify neuropathic pain	80.5	14.3	5.2
Tricyclics for AIDS pain	68.2	25.3	6.5
Family says: don’t tell	87.7	9.7	2.6
Tell prognosis in cancer	29.2	68.9	1.9
Define physician assisted suicide and euthanasia	83.8	16.2	0.0
Treat dyspnea	67.5	13.0	19.5
Diagnose hypercalcemia	55.8	35.8	8.4
Treat death rattle	20.8	62.3	16.9
No IV hydration for dying patient	38.3	26.6	35.1
Pronounce death	32.5	58.4	9.1
Identify normal grief	72.7	22.1	5.2

## Abbreviations

COPD: Chronic Obstructive Pulmonary Disease
EAPC: European association for palliative care
